# *Head & Face Medicine* reviewer acknowledgement 2015

**DOI:** 10.1186/s13005-016-0107-z

**Published:** 2016-02-04

**Authors:** Thomas Stamm, Ulrich Meyer, Hans Peter Wiesmann

**Affiliations:** University of Münster, Münster, Germany; University of Düsseldorf, Düsseldorf, Germany; Technische Universität Dresden, Dresden, Germany

## Abstract

The editors of *Head & Face Medicine* would like to thank all reviewers who have contributed their time and expertise to the journal in Volume 11 (2015).

**Swetha Acharya**

India

**Mehmet Cemal Akay**

Turkey

**Mohammad Khursheed Alam**

Bangladesh

**Carlos Eduardo Alcântara**

Brazil

**E A Al-Moraissi**

Yemen

**Isabel Andia**

Spain

**Thaschawee Arkachaisri**

Singapore

**Wolfgang H. Arnold**

Germany

**Athanasios Athanasiou**

United Arab Emirates

**Erica Avila**

United States

**Alberto Baldini**

Italy

**Boulos Bechara**

United States

**Maja Bendyk-Szeffer**

Poland

**Darpan Bhargava**

India

**Indraneel Bhattacharyya**

India

**Kerstin Bitter**

Germany

**Paolo Boffano**

Italy

**Ahmad Burhan**

Syria

**Sandra Bussadori**

Brazil

**Halil Çagatay**

Turkey

**Sirmahan Cakare**

Turkey

**Bing Cheng**

China

**Fábio Costa**

Brazil

**Adriano Crismani**

Austria

**Till Dammaschke**

Germany

**Gholamreza Danesh**

Germany

**Yannicke Dauphin**

France

**Christopher Davis**

United Kingdom

**Julio Cesar De Oliveira**

Brazil

**Luciana De-Marchi**

Brazil

**Ilze Dobele**

Latvia

**H El Nahass**

Egypt

**Aala Emara**

Egypt

**Seyda Ersahan**

Turkey

**Ahmad Eweida**

Egypt

**Ali Farahani**

United Kingdom

**Marijana Filipovska-Musanovic**

Bosnia And Herzegovina

**Roman Fischbach**

Germany

**Patrice Forget**

Belgium

**Edward Franek**

Poland

**Roland Frankenberger**

Germany

**Bernhard Frerich**

Germany

**Andreas R. Gantenbein**

Switzerland

**Robert Garcia**

France

**Olaide S Gbadebo**

Nigeria

**R Gedik**

Turkey

**Mehdi Gholami**

Iran

**Merve Goymen**

Turkey

**Chuanbin Guo**

China

**Yi Guo**

United States

**Laurent Guyot**

France

**Urban Hagg**

Hong Kong

**Mohammad Hajeer**

Syria

**Xin-Wei Han**

China

**K V S Hari Kumar**

India

**Ayman Hegab**

Egypt

**Andrew Hinson**

United States

**Ariane Hohoff**

Germany

**Akihiro Hosoya**

Japan

**Kazuhiko Imaizumi**

Japan

**Belma Isik Aslan**

Turkey

**Mario Isiordia**

Mexico

**Eren Isman**

Turkey

**Yusuf Izci**

Turkey

**Gundega Jakobsone**

Latvia

**Xin-Chun Jian**

United States

**Susanne Jung**

Germany

**Britta A. Jung**

Germany

**P Jungbluth**

Denmark

**Graça Justo**

Brazil

**Joseph Katz**

United States

**Hong-Seop Kho**

South Korea

**Tae-Woo Kim**

South Korea

**Gero Kinzinger**

Germany

**Christian Kirschneck**

Germany

**Michael Knoesel**

Germany

**Doruk Kocyigit**

Turkey

**Heike Korbmacher-Steiner**

Germany

**Fatih Mehmet Korkmaz**

Turkey

**Jolanta Kostrzewa-Janicka**

Poland

**Gerald Krennmair**

Antarctica

**Tae-Geon Kwon**

South Korea

**Manuel O Lagravere**

Canada

**Evangeli S Lampri**

Greece

**Rainer Laskawi**

Germany

**Guenter Lauer**

Germany

**Uilyong Lee**

South Korea

**Carsten Lippold**

Germany

**Di-Qing Luo**

China

**Robert Bruce Macintosh**

Micronesia

**Manuele Mancini**

Italy

**Giuditta Mannelli**

Italy

**Peter Maurer**

Germany

**Edward Mbewe**

Zambia

**Margaret Mclaughlin-Drubin**

United States

**Francisco Javier Medina Fernández**

Spain

**Giuseppe Monaco**

Italy

**Urs D. A. Müller-Richter**

Germany

**Takahito Nakajima**

Japan

**Jörg Neunzehn**

Germany

**Ruba Odeh**

United Kingdom

**Fatih Oghan**

Turkey

**Jooyoung Ohe**

South Korea

**Sergio Olate**

Chile

**Michelle Ommerborn**

Germany

**Siti Adibah Othman**

Malaysia

**Mario Pagnoni**

Italy

**Vitor Panhóca**

Brazil

**Chan Hum Park**

South Korea

**Jae Park**

United States

**Anne-Lise Poirrier**

Belgium

**Michal Polguj**

Poland

**Amaury Pozos**

Mexico

**Vahid Rakhshan**

Iran

**Ksenija Rener-Sitar**

Slovenia

**Michael Richards**

United States

**Matthias Roggendorf**

Germany

**Jianping Ruan**

China

**Parappa Sajjan**

India

**Atsushi Sano**

Japan

**Kes Schroer**

United States

**Matthias Schulz**

Germany

**Falk Schwendicke**

Germany

**Robin Seeberger**

Germany

**Ahmet-Ercan Sekerci**

Turkey

**Emmanuel Joao Nogueira Leal Silva**

Brazil

**Paulo Goberlanio De Barros Silva**

Brazil

**Ralf Smeets**

Germany

**Janet Southerland**

United States

**Derek Steinbacher**

United States

**Markus Stenner**

Germany

**Lisha Sun**

China

**Enes Tan**

Turkey

**Bien Keem Tan**

Singapore

**Ichih Tan**

United States

**Der-Cherng Tarng**

Taiwan

**Umut Tekin**

Turkey

**Afshin Teymoortash**

Germany

**Weidong Tian**

China

**Ji Tong**

China

**Florian Uecker**

Germany

**Jop Verweij**

Netherlands

**Joan Viciano**

Italy

**Rick Visser**

Spain

**J Von Bremen**

Germany

**Wen-Hung Wang**

Taiwan

**Nuray Yilmaz Altintas**

Turkey

**Shengyuan Yu**

China

**Yehuda Zadik**

Israel

**Mohammad Zandi**

Iran

**Yu Zhou**

China

**Thomas Ziebura**

Germany

